# The study of bone healing after endodontic microsurgery using cone beam computed tomography: A retrospective cohort study

**DOI:** 10.4317/jced.58354

**Published:** 2022-08-01

**Authors:** Amparo Ramis-Alario, Beatriz Tarazona-Álvarez, Miguel Peñarrocha-Diago, David Soto-Peñaloza, María Peñarrocha-Diago, David Peñarrocha-Oltra

**Affiliations:** 1 DDS, MS. Master in Oral Surgery and Implantology, Valencia University Medical and Dental School, Valencia, Spain; 2 DDS, PhD. Postdoctoral Associate Professor, Department of Stomatology, Valencia University Medical and Dental School, Valencia, Spain; 3 MD, PhD. Chairman of Oral Surgery and Director of the Master in Oral Surgery and Implantology, Valencia University Medical and Dental School, Valencia, Spain; 4 MD, DDS, MS, PhD. Full Professor of Oral Surgery, Department of Stomatology, Valencia University Medical and Dental School, Valencia, Spain

## Abstract

**Background:**

The main aims of this study were to evaluate mean volume reduction, radiographic healing rate and healing outcome two years after endodontic microsurgery. The effects of certain preoperative clinical factors upon preoperative volume, volumetric changes and healing outcome were also studied.

**Material and Methods:**

A clinical database was searched for patients who had undergone endodontic microsurgery and with the availability of a cone beam computed tomography (CBCT) scan preoperatively and after a control period. Volumetric analysis of the periapical area was made to assess volumetric reduction. The modified Penn 3D criteria were applied. The relationship between preoperative volume, volumetric reduction and healing outcome and certain preoperative factors was also studied.

**Results:**

Fifty-seven cases were evaluated. Initially, the bone cavities had a median volume of 163.2 mm3, and this volume decreased by 147.7 mm3 after treatment, with a radiographic healing rate of 6.2 mm3 per month. After applying the modified Penn 3D Criteria, 53 cases were classified as successful healing (93%). Regarding the influence of the different preoperative factors, patient age and sex, dental arch and cortical bone significantly influenced preoperative volume, while only the dental arch exerted a significant influence upon volumetric changes and preoperative symptoms on healing outcome.

**Conclusions:**

The CBCT data evidenced a significant volume reduction of 79.1%, with a monthly volume reduction rate of 6.2 mm3. The success rate obtained was 93%. Patient age and sex, dental arch and cortical bone influenced preoperative volume, tooth type had an impact upon the volumetric changes, and the preoperative symptoms influenced healing outcome.

** Key words:**Cone beam computed tomography, endodontic microsurgery, healing, lesion volume, prognostic factors.

## Introduction

As a result of the latest technical advances in endodontic microsurgery, the success rates of such treatment are now very high, in the range of 90% (1,2) and define such surgery as a reliable patient management option. These advances include preoperative study involving cone beam computed tomography (CBCT), the use of high magnification, ultrasonic root-end preparation, and the use of biocompatible root-end filling materials.

Cone beam computed tomography has demonstrated great diagnostic sensitivity prior to endodontic microsurgery (3-5), and is very useful for monitoring patients after such surgery (6,7) or following endodontic retreatment (7). Nowadays CBCT is widely accepted for the diagnosis of periapical disease as well as for planning endodontic microsurgery, but it is not regarded as the gold standard for monitoring these endodontic cases over time (8). In some cases, as when a tooth is intended to be used as an abutment for a prosthesis, it is important to be sure about the outcome of endodontic microsurgery or endodontic retreatment. According to studies based on periapical radiographs, the indicated follow-up time to ensure healing after periapical surgery is one year (9-11). However, considering the superiority of CBCT in the diagnosis of these lesions, it would be useful to assess the course of healing over time using this imaging technique. With regard to the size of the periapical lesion prior to endodontic microsurgery, some studies report that the areas measured by CBCT are larger than those measured by two-dimensional radiographs (6,12). On the other hand, some authors have observed no significant differences between two- (2D) and three-dimensional (3D) imaging-based diagnoses as far as lesion size is concerned (13,14). The relationship between other preoperative factors (i.e., the presence of pain, probing depth, patient age and sex, etc.) and the surgical outcomes has also been studied, and there are conclusions both in favor and against their influence upon the results of surgery (7,15,16).

The present study was carried out to evaluate the mean volume reduction of the periapical lesions two years after endodontic surgery, the radiographic healing rate and the healing outcome after endodontic microsurgery, based on the use of CBCT. The effects of certain preoperative clinical factors upon preoperative volume, volumetric changes and healing outcome were also studied.

## Material and Methods

-Study design and patient selection

A retrospective cohort study was carried out in the Unit of Oral Surgery (Department of Stomatology) of the University of Valencia (Valencia, Spain), following approval by the Research Ethics Committee of the University of Valencia (Ref. nº. H1523379927800). The study sample was selected on a consecutive basis from a population of patients in the Department of Stomatology who had undergone endodontic microsurgery between January 2015 and January 2018. For statistical reasons, only one tooth per patient was included. Each patient signed a consent form based on the principles of the Declaration of Helsinki (2013), accepting participation in the study and the performance of CBCT controls. This study is presented following the STROBE guidelines (17).

-Inclusion and exclusion criteria

The following inclusion criteria were applied:

1. Patients subjected to primary endodontic microsurgery with the use of an endoscope and ultrasonic instruments, and employing mineral trioxide aggregate (MTA) for retrograde filling.

2. Availability of a CBCT scan performed two years after the surgery.

The following exclusion criteria were applied:

1. Endodontic surgery involving bone grafting or the use of barrier materials.

2. Patients failing to report to the follow-up visits.

-Surgical procedures

Modern surgical techniques were used in all patients, and all the operations were carried out by the same oral surgeon (M.P.D.) (18). High magnification with a rigid endoscope (Karl Storz-Endoskope, Tuttlingen, Germany) was used, with ultrasonic preparation (Piezon® Master 400, EMS®, Electro Medical Systems S.A, Switzerland) and root-end filling with MTA (ProRoot MTA White, Dentsply Tulsa Dental, Tulsa, OK, USA).

-Radiographic evaluation

All the CBCT images were obtained using the Planmeca ProMax 3D Classic CBCT unit (Planmeca, Helsinki, Finland) with the patient in the sitting position. Image processing was carried out with Planmeca Romexis® software (version 4.5.2., Planmeca). A limited field of view (40 x 40 mm) with a 12-second duration and settings of 90 kV, 8 mA and voxel size 0.15 mm were used. All the CBCT scans were devoid of personal identifying data.

**Figure 1:**
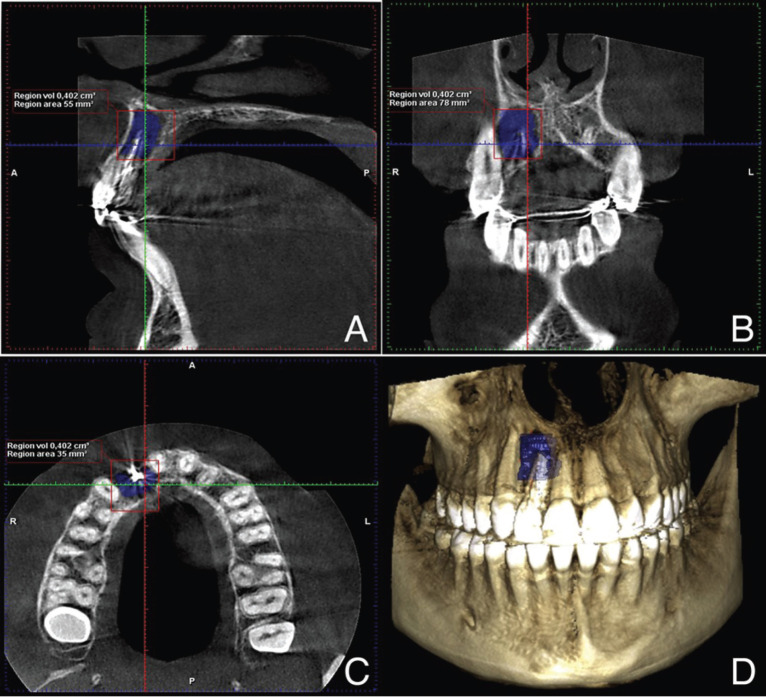
Volumetric assessment of a periapical lesion of a lateral upper right incisor using CBCT. A) Coronal plane. B) Sagittal plane. C) Axial view. D) 3D image of the traced lesion.

Lesion volume was measured both pre- and postoperatively by CBCT using the same software. The area was traced manually, measuring from the first to the last slice in which the lesion was observed. Then the program automatically calculated the total outlined volume. In anterior teeth (canines and incisors), tracing was performed from axial sections, because due to their vestibular tilt, this plane registered all the area better. In posterior teeth (molars and premolars), tracing was performed from coronal sections because, due to interpositioning of the maxillary sinus, these allowed better visualization of the lesion. If a multiple-root tooth had more than one periapical area, the individual defect volumes were added and counted as one (6). An example of the volumetric assessment of the periapical lesions is shown in Figure 1.

**Figure 2:**
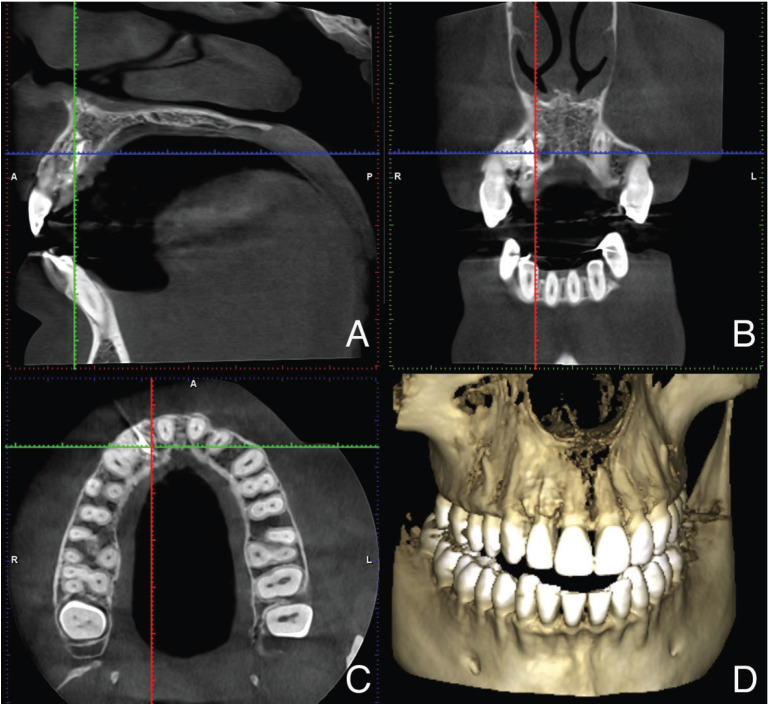
The same case as in Figure 1, in the control period. Volume reduction was 100% (counted as 0 mm3). A) Coronal plane. B) Sagittal plane. C) Axial view. D) 3D image of the traced lesion.

The program automatically transformed the number of voxels measured into mm3. In the postoperative CBCT scan, any radiolucent volumes with less than twice the width of the periodontal ligament were assessed as 0 mm3 (6) (Fig. 2).

These measurements were made jointly by two pre-calibrated investigators (A.R.A. and D.P.O.). Both examiners discussed and reached consensus on borderline designations. Previously, in order to assess the validity of both examiners, 10 CBCT scans not involved in the final sample were evaluated independently by these two examiners.

All images were evaluated on a 21.5” monitor (iMac; Apple, Cupertino, CA, USA) with a screen resolution of 4096 x 2304 pixels and located in a quiet room with subdued lighting.

-Data collection

Lesion volume reduction: initial lesion volume – final lesion volume (expressed as a percentage).

Radiographic healing rate: lesion volume reduction / time elapsed between surgery and control CBCT.

**Figure 3:**
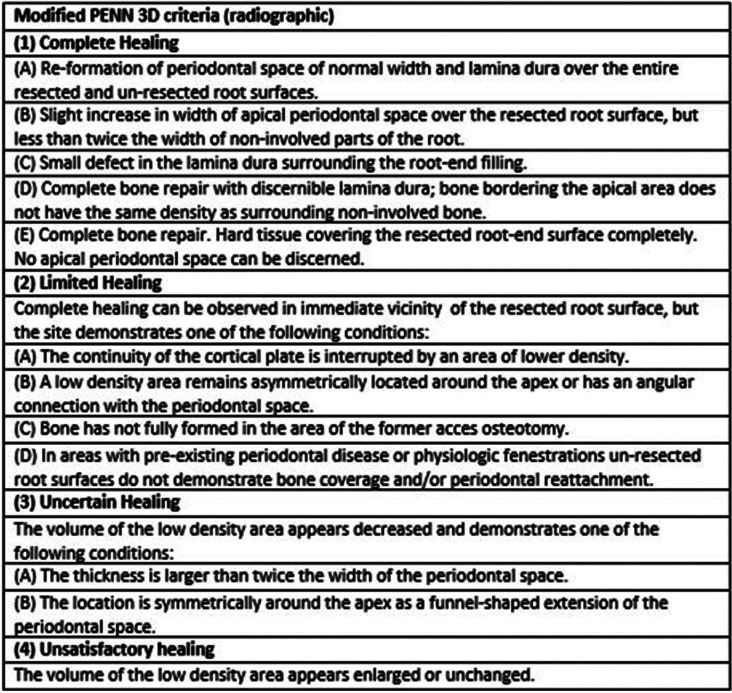
Modified Penn 3D criteria.

Healing assessment: the modified Penn 3D criteria were used to assess the results of endodontic microsurgery (19), classifying healing as complete, limited, uncertain or unsatisfactory (Fig. 3). This classification was made independently by two examiners (A.R.A. and D.P.O). Disagreement between the examiners was resolved by discussion with a third advisor (D.S.P). The study unit was considered to be the tooth, and multiple-root teeth were classified according to the worst-appearing root (15). Outcomes were dichotomized as either success (complete or limited healing) or failure (uncertain or unsatisfactory healing) (6).

-Preoperative clinical variables 

The following variables were collected: preoperative volume, patient age and sex, smoking, symptoms and signs, type of tooth, dental arch, the presence of intraradicular posts, bone crest level and cortical fenestration. The criteria of von Arx and Kurt were used to assess the patient symptoms and signs (20).

-Statistical analysis

Inter-examiner agreement in the calculation of lesion volume was assessed with the intraclass correlation coefficient (ICC). In turn, inter-examiner agreement in assessing healing outcome was evaluated with the Cohen kappa statistic. Both agreements were interpreted respectively by the Fleiss scale (21) and the Landis and Koch scale (22).

With regard to analysis of the variables, the Student t-test was used to evaluate the changes in volume distribution from pre- to post-endodontic microsurgery. In order to relate the preoperative clinical parameters to initial volume, average volumetric reduction and healing outcome, use was made of the Mann-Whitney U-test (MW), Kruskal Wallis test (KW), Fisher exact test and Spearman’s correlation coefficient. Patient age was dichotomized as either ≤ or > 45 years, as in a previous study (23), only to assess volumetric reduction. The type of tooth was defined as incisor, canine, premolar or molar, and the dental arch was also considered. The bone crest level was measured as either < or ≥ 3 mm from the cementoenamel junction to the bone crest.

Statistical significance was considered for *p* < 0.05. The SPSS version 15.0 statistical package (Chicago, IL, USA) was used throughout.

## Results

Of the 298 patients subjected to endodontic microsurgery between January 2015 and January 2018, 15 had undergone previous endodontic microsurgery, 12 had teeth filled with materials other than MTA, 154 patients did not accept a control CBCT scan after two years or the control CBCT scan was not performed two years after endodontic microsurgery, 35 received bone grafting or barrier materials during surgery, and 25 patients were lost to follow-up. A total of 57 patients met the inclusion criteria, representing a total of 57 operated teeth. The characteristics of the study sample are described in Table 1.

**Table 1:**
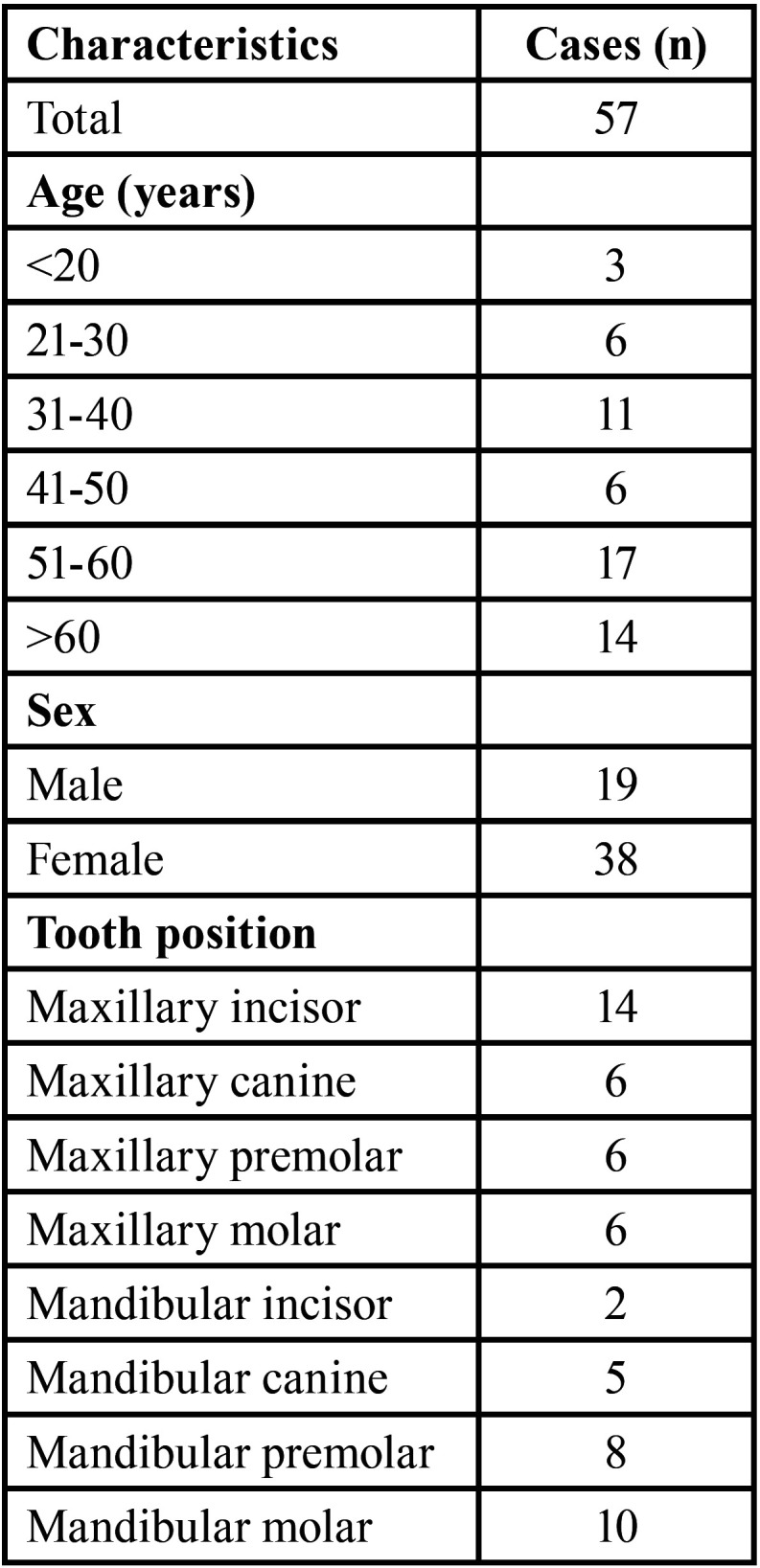
Distribution of the evaluated cases.

The ICC for the volume measurements was 0.857 (95% confidence interval [95%CI] 0.822-0.885), evidencing excellent agreement between the two examiners. In turn, the Kappa index for healing assessment was 89.5%, which was rated as very good in the Landis and Koch evaluation. Preoperatively, the mean absolute lesion volume was 163.2 mm3 (n=57, standard deviation [SD] ± 288.4 mm3; range 11-1373 mm3), with a median of 62.0 mm3. In turn, the mean absolute lesion volume in the postoperative images was 15.5 mm3 (n=57, SD ± 37.4 mm3; range 0-227 mm3), with a median of 0 mm3. The lesions decreased an average of 147.7 mm3 (SD ± 282.1 mm3). In terms of percentages, the mean volumetric change was 79.1% (median = 100%). In 31 cases the volume reduction was 100%; in 6 cases the mean reduction was over 90%; in 12 cases the reduction was less than 90% but more than 50%; in four cases the reduction was between 50% and 0%; and in four cases an increase in lesion volume was recorded. These changes in volume were statistically significant (*p*<0.001, paired t-test). The four lesions that increased in volume all presented a preoperative volume below the median - the maximum being 56 mm3. The mean radiographic healing rate was 6.2 mm3/month (SD ± 12.6 mm3).

**Figure 4:**
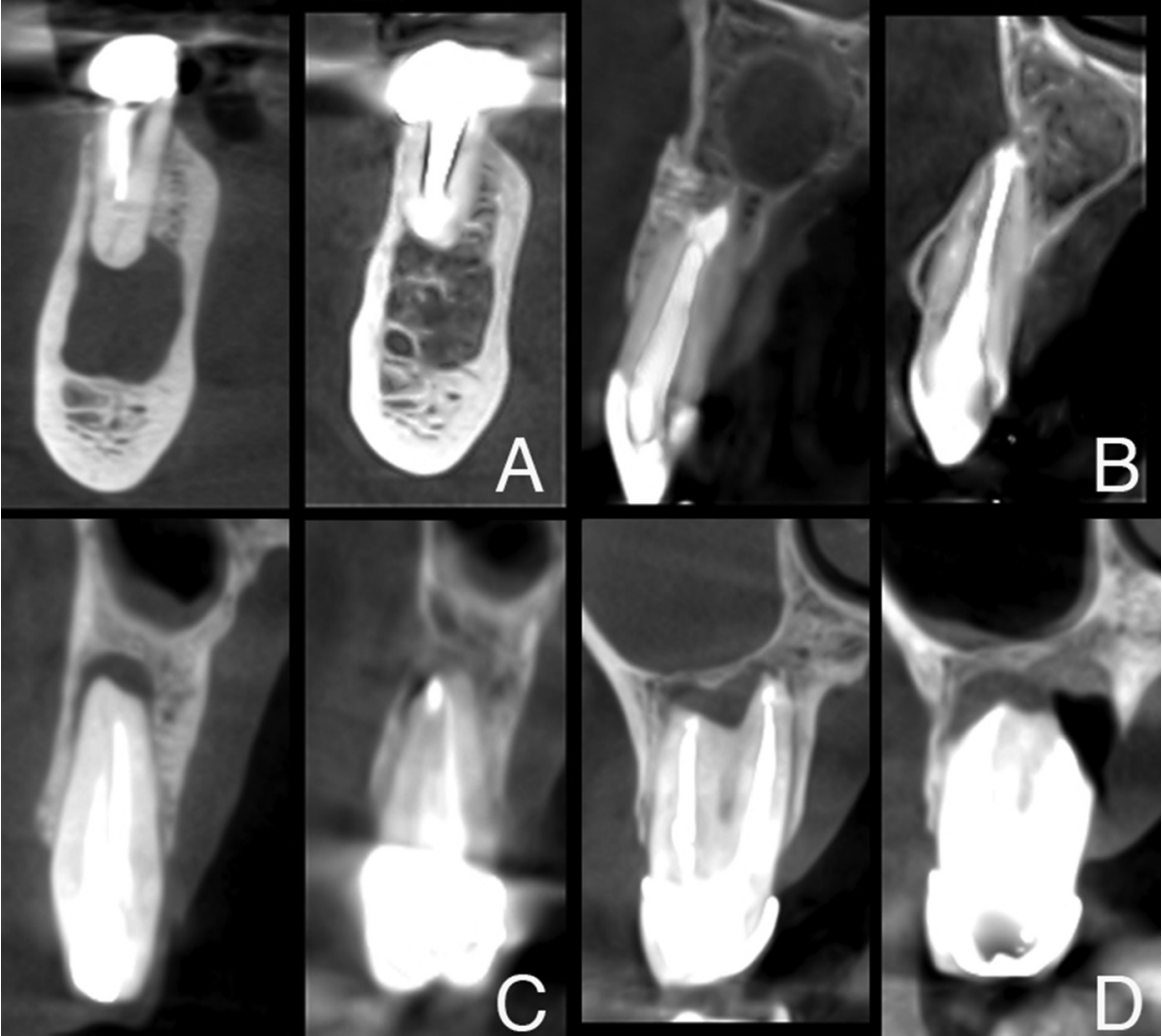
Healing of periapical lesions after endodontic microsurgery according to the modified Penn 3D criteria. A) Complete healing. B) Limited healing. C) Uncertain healing. D) Unsatisfactory healing.

In the assessment of healing outcome, and after discussion of 6 cases in which the examiners initially failed to agree, a total of 37 teeth were classified as complete healing, 16 teeth as limited healing, one tooth as uncertain healing, and three teeth as unsatisfactory healing (Fig. 4). Thus, the final classification was: 53 teeth classified as success and four teeth classified as failure. The overall success rate was therefore 93% (53/57).

Preoperative volume was significantly influenced by patient age and sex, the dental arch and the cortical bone conditions. Larger lesions were seen in younger patients (*p*=0.006, r=-0.36), males (*p*=0.031, MW), maxillary teeth (*p*=0.014, MW) and in lesions with cortical fenestration (*p*=0.002, MW). Only the type of tooth significantly influenced the volumetric changes, with greater volume reduction being recorded in incisors, canines and premolars (*p*=0.036, KW). The influence of crestal bone level and cortical perforation upon the volume changes tended towards clinical significance. Only the preoperative symptoms had a significant impact upon the healing outcome of endodontic microsurgery (*p*=0.032, KW) (Table 2).

**Table 2:**
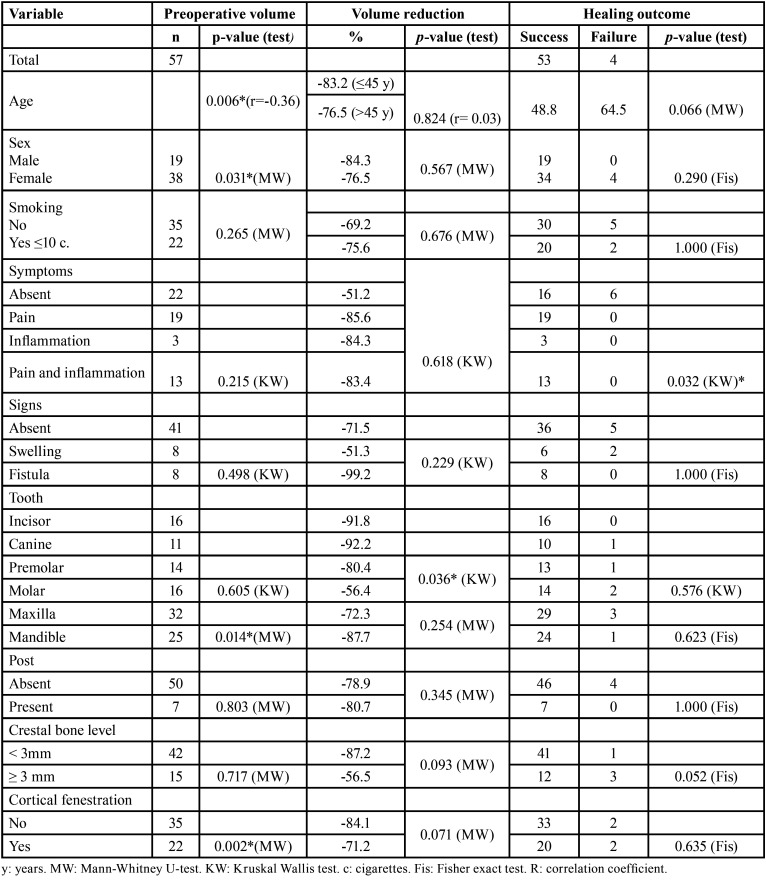
Analysis of the influence of the preoperative parameters upon preoperative volume, volume reduction and healing outcome.

## Discussion

The present retrospective cohort study analyzed the decrease in volume, the radiographic healing rate and healing outcome of a group of teeth with periapical disease and subjected to endodontic microsurgical treatment, based on the CBCT findings. The duration of follow-up was two years, which can be accepted as sufficient according to the literature (6,7). The lesions decreased in volume an average of 147.7 mm3, from a mean preoperative value of 163.2 mm3 to a postoperative mean volume of 15.5 mm3, corresponding to a mean healing rate of 6.2 mm3 per month. Of the 57 cases with periapical disease, 53 were considered to represent successful healing.

In analyzing the healing rate, it may be useful to know when the implicated tooth can be used for other purposes, if necessary. The best way to establish this is via biopsy, though this approach is not feasible in routine practice; a CBCT study therefore could be a good option. Nevertheless, the healing rate could be underestimated in this case, since the CBCT scan may have been performed some time after healing completion. The healing rate could only be accurately calculated if CBCT scans were made each month, though such a practice would not be justified.

The mean reduction in volume of the periapical areas in our study was 79.1%. This differs from the findings of Curtis *et al*. (7), who reported an average volumetric change of 95%, but is similar to the percentage reported by Schloss *et al*. (83.7%) (6), though these authors reported 38 cases in which the volume reduction was 100%, 7 cases with a reduction of > 90%, two cases with < 90%, and four cases in which the reduction was < 0%. These cases of healing with volume reductions of > 90% were more numerous than in our series. The differences among the studies may be due to the fact that the evaluation times after surgery were longer than in our study, reaching 48 months in the study by Curtis *et al*. and 37 months in that the publication of Schloss *et al*.

In the present study, a volume reduction was observed in 53 out of 57 teeth, and according to the modified Penn 3D criteria, 93% of them healed successfully. These outcomes are similar to those reported by other studies in recent years. Schloss *et al*. (6), who used the same healing criteria, recorded a successful healing rate of 92.2%. In turn, Parmar *et al*. (24) carried out a study involving 30 through-and-through periapical lesions divided into two equal groups - one group regenerated with a resorbable collagen membrane and the other group without regeneration. Both groups yielded a healing success rate of 87%. This lower success rate could have been due to the complexity of their sample.

With respect to the initial volume of the periapical areas, many studies have explored possible relations between the initial size of the lesion and healing assessment after endodontic surgery. Some authors consider initial lesion size to influence the prognosis of surgery (16,25), while other have observed no such relationship (26). We divided our sample based on the median initial volume (62 mm3), which was quite similar to that of Kreisler *et al*. (25) with a median of 60 mm3; of the four cases classified as failures, none were larger than the median: no relationship was therefore observed between initial volume and the final outcome.

Regarding the influence of the preoperative variables upon the preoperative volume, Cheung *et al*. (27) observed differences between the two dental arches in their CBCT evaluation of the preoperative volume, but did not specify whether these differences reached statistical significance. Lemagner *et al*. (28) in turn carried out a study on teeth with an without endodontic treatment, and concluded that there was no relationship between the dimension of the apical bone defects and patient sex or tooth position (mandibular or maxillary). In this study, only the type of tooth was seen to influence mean volume reduction, with a lesser decrease in posterior teeth. The explanation for this is that in the case of molars - particularly lower molars and the palatal root of the upper molars - the amount of bone that needs to be removed during surgery is greater. It is therefore likely that after two years of follow-up, the bone has not completely healed. Öğütlü *et al*. (29) also reported poorer healing in posterior teeth at 6 months after periapical surgery, compared to anterior teeth.

With regard to the relationship between the preoperative parameters and the healing outcomes, significant differences were observed in relation to the preoperative symptoms, with more failures among the patients without such symptoms – though this may be a casual finding due to the few failed cases in our series. Von Arx *et al*. (15) conducted a 5-year longitudinal study analyzing a series of preoperative variables and their influence upon healing outcome. Their findings coincided with our own data in relation to many of the analyzed preoperative parameters, though the mentioned authors recorded significant differences in crestal bone level, while in our series differences were found between crestal bone level and volume reduction, though without reaching statistical significance. Kim *et al*. (16) also studied the destruction of cortical bone, and recorded no impact of this variable upon the final outcome. Curtis *et al*. (7) analyzed the influence of a number of preoperative and intraoperative variables, and although they did not all match our own evaluated factors, the mentioned authors recorded no association for any of the examined parameters.

With regard to the strengths and limitations of the present study, mention must be made of the homogeneity of the surgical procedure (e.g., the use of a rigid endoscope, root-end filling material, the hemostatic technique employed, and the radiographic techniques) by a surgeon with expertise in endodontic microsurgery. The data of most of the included patients were previously registered using standardized forms, and most potential confounders were accounted for statistically to deal with the selection bias inherent to the retrospective study design. On the other hand, the greater the volumes of the periapical areas, the greater the margin of error that can be made in the measurement. In this sense, duplicate assessment by precalibrated examiners was carried out to deal with bias in the measurement of outcomes. In short, the stringent inclusion criteria ensured a clinically valuable and representative study cohort. Further studies are needed to confirm the reproducibility of our findings. The data obtained are useful for surgeons and dental practitioners in rehabilitating teeth subjected to endodontic microsurgery.

## Conclusions

The periapical area exhibited a significant volume reduction of 79.1% two years after endodontic microsurgery. According to follow-up with CBCT, the monthly volume reduction rate was found to be 6.2 mm3; this can be useful for certain prosthetic situations, contributing to treatment planning. The recorded success rate was 93%, which is consistent with the existing scientific evidence. The magnitude of the preoperative volume was influenced by patient age and sex, the dental arch involved, and the cortical bone conditions. The type of tooth influenced the volumetric changes, while the clinical symptoms had an impact upon healing assessment.
